# Bis(2,5-dihydroxy­benzoato-κ*O*)bis­(1,10-phenathroline-κ^2^ *N*,*N*′)cadmium(II) 1.25-hydrate

**DOI:** 10.1107/S1600536808018126

**Published:** 2008-06-19

**Authors:** Bing-Yu Zhang, Jing-Jing Nie, Duan-Jun Xu

**Affiliations:** a Department of Chemistry, Zhejiang University, People’s Republic of China

## Abstract

In the crystal structure of the title compound, [Cd(C_7_H_5_O_4_)_2_(C_12_H_8_N_2_)_2_]·1.25H_2_O, the Cd^2+^ cation is coordinated by two phenanthroline (phen) mol­ecules and two 2,5-dihydroxy­benzoate (dhba) anions in a distorted octa­hedral geometry. The centroid–centroid distances of 3.809 (2) and 3.680 (2) Å between nearly parallel pyridine rings of the phen ligands and the benzene rings of dhba anions indicate that the dhba anions are involved in π–π stacking in the crystal structure. The face-to-face separation of 3.35 (3) Å between parallel phen ring systems also suggests π–π stacking between adjacent complex mol­ecules. The crystal structure contains extensive O—H⋯O and C—H⋯O hydrogen bonding.

## Related literature

For general background, see: Su & Xu (2004 ▶); Li *et al.* (2005 ▶). For a related structure, see: Huang *et al.* (2006 ▶).
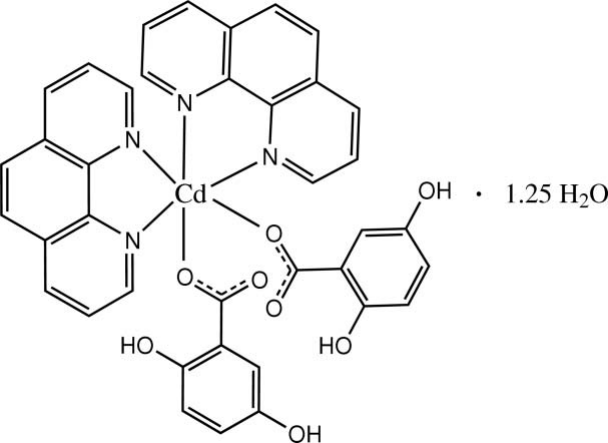


## Experimental

### 

#### Crystal data

[Cd(C_7_H_5_O_4_)_2_(C_12_H_8_N_2_)_2_]·1.25H_2_O
*M* *_r_* = 801.55
Monoclinic, 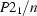

*a* = 10.8992 (18) Å
*b* = 27.300 (2) Å
*c* = 11.4218 (12) Å
β = 93.700 (6)°
*V* = 3391.5 (7) Å^3^
*Z* = 4
Mo *K*α radiation
μ = 0.71 mm^−1^
*T* = 295 (2) K
0.20 × 0.16 × 0.12 mm

#### Data collection

Rigaku R-AXIS RAPID IP diffractometer
Absorption correction: multi-scan (*ABSCOR*; Higashi, 1995 ▶) *T*_min_ = 0.875, *T*_max_ = 0.928
25199 measured reflections
6639 independent reflections
4509 reflections with *I* > 2σ(*I*)
*R*_int_ = 0.064

#### Refinement

*R*[*F* ^2^ > 2σ(*F* ^2^)] = 0.042
*wR*(*F* ^2^) = 0.092
*S* = 1.03
6639 reflections
478 parameters
H-atom parameters constrained
Δρ_max_ = 0.53 e Å^−3^
Δρ_min_ = −0.51 e Å^−3^

### 

Data collection: *PROCESS-AUTO* (Rigaku, 1998 ▶); cell refinement: *PROCESS-AUTO*; data reduction: *CrystalStructure* (Rigaku/MSC, 2002 ▶); program(s) used to solve structure: *SIR92* (Altomare *et al.*, 1993 ▶); program(s) used to refine structure: *SHELXL97* (Sheldrick, 2008 ▶); molecular graphics: *ORTEP-3* (Farrugia, 1997 ▶); software used to prepare material for publication: *WinGX* (Farrugia, 1999 ▶).

## Supplementary Material

Crystal structure: contains datablocks I, global. DOI: 10.1107/S1600536808018126/rk2092sup1.cif

Structure factors: contains datablocks I. DOI: 10.1107/S1600536808018126/rk2092Isup2.hkl

Additional supplementary materials:  crystallographic information; 3D view; checkCIF report

## Figures and Tables

**Table 1:** Hydrogen-bond geometry (Å, °)

*D*—H⋯*A*	*D*—H	H⋯*A*	*D*⋯*A*	*D*—H⋯*A*
O1*W*—H1*A*⋯O1	0.94	2.09	2.974 (6)	155
O1*W*—H1*B*⋯O6	0.92	2.03	2.892 (6)	155
O2*W*—H2*A*⋯O2	0.88	1.99	2.869 (18)	175
O2*W*—H2*B*⋯O8^i^	0.86	2.42	3.28 (2)	173
O3—H3*A*⋯O2	0.82	1.81	2.540 (3)	147
O4—H4*A*⋯O7^ii^	0.82	2.09	2.877 (3)	160
O7—H7*A*⋯O6	0.82	1.82	2.546 (3)	147
O8—H8*A*⋯O3^iii^	0.82	2.10	2.917 (4)	171
C23—H23⋯O1*W*^iv^	0.93	2.49	3.339 (6)	153
C25—H25⋯O6^v^	0.93	2.50	3.285 (5)	143
C30—H30⋯O1	0.93	2.56	3.155 (5)	122
C33—H33⋯O5	0.93	2.50	3.105 (5)	123
C38—H38⋯O2^vi^	0.93	2.36	3.182 (5)	147
C42—H42⋯O4^vii^	0.93	2.58	3.231 (5)	127

Symmetry codes: (i) 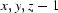; (ii) 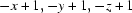; (iii) 
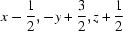
; (iv) 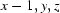; (v) 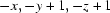; (vi) 
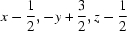
; (vii) 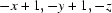.

**Fig. 1.**
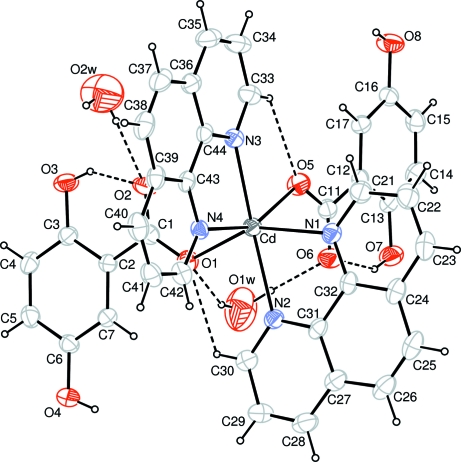
The molecular structure of the title compound with the numbering scheme. The displacement ellipsoids are drawn at 40% probability level. H atoms are presented as a small spheres of arbitrary radius. Dashed lines indicate hydrogen bonding.

**Fig. 2.**
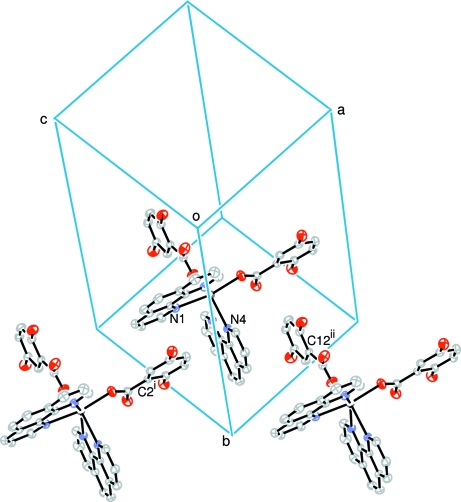
A diagram showing π-π stacking between phen and dhba (symmetry codes: (i) x-1, y, z; (ii) x, y, z-1).

**Fig. 3.**
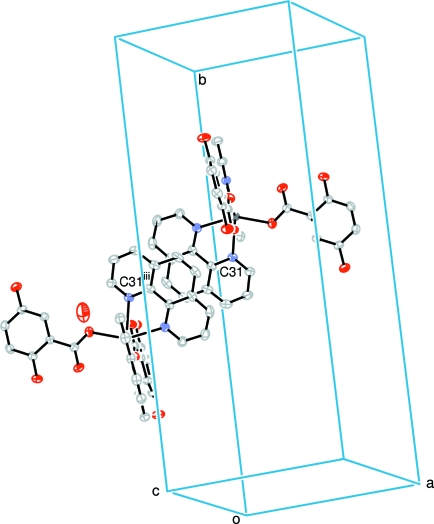
A diagram showing π-π stacking between phen ligands (symmetry code: (iii) -x, 1-y, 1-z).

## supplementary crystallographic information

### Comment

As part of investigation on the nature of π-π stacking between aromatic rings (Su & Xu, 2004; Li *et al.*, 2005), the title complex recently has been prepared and its crystal structure is reported here.

The molecular structure of the title compound is shown on Fig. 1. The Cd^2+^ cation is coordinated by two phenanthroline (phen) ligands and two 2,5-dihydroxybenzoate (dhba) anions with a distorted octahedral geometry. The centroid-to-centroid distance of 3.809 (2)Å between nearly parallel N1-pyridine and C2^i^-benzene rings (dihedral angle 4.89 (17)°; symmetry code: (i) *x-1*, *y*, *z*) and the centroid-to-centroid distance of 3.680 (2)Å between nearly parallel N4-pyridine and C12^ii^-benzene rings (dihedral angle 5.33 (11)°; symmetry code: (ii) *x*, *y*, *z*-1) indicate that dhba anions are involved in π-π stacking in the crystal structure (Fig. 2), which agrees with the situation found in the 3,5-dihydroxybenzoate complex of Cu^2+^ (Huang *et al.*, 2006). The face-to-face separation of 3.35 (3)Å suggests the existence of π-π stacking between parallel C31-phen and C31^iii^-phen ring systems (Fig. 3) (symmetry code: (iii) -*x*, 1-*y*, 1-*z*). The crystal structure contains extensive O–H···O and C–H···O hydrogen bonding (Table 1).

### Experimental

Cd(NO_3_)_2_^.^4H_2_O (0.31 g, 1 mmol), dhba (0.31 g, 2 mmol), phen (0.36 g, 2 mmol) and Na_2_CO_3_ (0.10 g, 1 mmol) were dissolved in a water-ethanol mixture (20 ml, 2:1). The solution was refluxed for 2 h. After cooling to room temperature the solution was filtered. Single crystals of the title compound were obtained from the filtrate after 4 weeks.

### Refinement

The site occupancy factor of the O2W water molecule was initially refined and converged to 0.28, and fixed as 0.25 at final cycles of refinemens. Water H atoms were placed in chemical sensible positions and refined in riding mode with *U*_iso_(H) = 1.5*U*_eq_(O). Other H atoms were placed in calculated positions with C–H = 0.93Å and O–H = 0.82Å, and refined in riding mode with *U*_iso_(H) = 1.2*U*_eq_(C) or 1.5*U*_eq_U(O).

### Crystal data

**Table:**

[Cd(C_7_H_5_O_4_)_2_(C_12_H_8_N_2_)_2_]·1.25H_2_O	*F*_000_ = 1626
*M**_r_* = 801.55	*D*_x_ = 1.570 Mg m^−^^3^
Monoclinic, *P*2_1_/*n*	Mo *K*α radiation λ = 0.71073 Å
Hall symbol: -P 2yn	Cell parameters from 6929 reflections
*a* = 10.8992 (18) Å	θ = 2.2–25.5º
*b* = 27.300 (2) Å	µ = 0.71 mm^−^^1^
*c* = 11.4218 (12) Å	*T* = 295 (2) K
β = 93.700 (6)º	Prism, colourless
*V* = 3391.5 (7) Å^3^	0.20 × 0.16 × 0.12 mm
*Z* = 4	

### Data collection

**Table:**

Rigaku R-AXIS RAPID IP diffractometer	6639 independent reflections
Radiation source: Fine-focus sealed tube	4509 reflections with *I* > 2σ(*I*)
Monochromator: Graphite	*R*_int_ = 0.064
Detector resolution: 10.0 pixels mm^-1^	θ_max_ = 26.0º
*T* = 295(2) K	θ_min_ = 1.5º
ω scans	*h* = −13→13
Absorption correction: multi-scan(ABSCOR; Higashi, 1995)	*k* = −33→31
*T*_min_ = 0.875, *T*_max_ = 0.928	*l* = −8→14
25199 measured reflections	

### Refinement

**Table:**

Refinement on *F*^2^	Secondary atom site location: Difmap
Least-squares matrix: Full	Hydrogen site location: Geom
*R*[*F*^2^ > 2σ(*F*^2^)] = 0.042	H-atom parameters constrained
*wR*(*F*^2^) = 0.092	*w* = 1/[σ^2^(*F*_o_^2^) + (0.0349*P*)^2^] where *P* = (*F*_o_^2^ + 2*F*_c_^2^)/3
*S* = 1.03	(Δ/σ)_max_ = 0.001
6639 reflections	Δρ_max_ = 0.53 e Å^−^^3^
478 parameters	Δρ_min_ = −0.51 e Å^−^^3^
Primary atom site location: Direct	Extinction correction: none

### Special details

**Table:**

Geometry. All s.u.'s (except the s.u. in the dihedral angle between two l.s. planes) are estimated using the full covariance matrix. The cell s.u.'s are taken into account individually in the estimation of s.u.'s in distances, angles and torsion angles; correlations between s.u.'s in cell parameters are only used when they are defined by crystal symmetry. An approximate (isotropic) treatment of cell s.u.'s is used for estimating s.u.'s involving l.s. planes.
Refinement. Refinement of *F*^2^ against ALL reflections. The weighted *R*-factor *wR* and goodness of fit *S* are based on *F*^2^, conventional *R*-factors *R* are based on *F*, with *F* set to zero for negative *F*^2^. The threshold expression of *F*^2^ > σ(*F*^2^) is used only for calculating *R*-factors(gt) *etc*. and is not relevant to the choice of reflections for refinement. *R*-factors based on *F*^2^ are statistically about twice as large as those based on *F*, and *R*-factors based on ALL data will be even larger.

### Fractional atomic coordinates and isotropic or equivalent isotropic displacement parameters (Å^2^)

**Table:**

	*x*	*y*	*z*	*U*_iso_*/*U*_eq_	Occ. (<1)
Cd	0.27239 (2)	0.627675 (9)	0.32033 (2)	0.03808 (10)	
N1	0.0736 (3)	0.61272 (10)	0.3827 (3)	0.0437 (8)	
N2	0.2176 (3)	0.54243 (10)	0.2903 (3)	0.0426 (7)	
N3	0.2325 (3)	0.71386 (10)	0.2909 (3)	0.0409 (7)	
N4	0.1908 (2)	0.64287 (10)	0.1245 (3)	0.0373 (7)	
O1	0.4520 (2)	0.60327 (9)	0.2586 (2)	0.0554 (7)	
O2	0.5248 (2)	0.67858 (9)	0.2365 (2)	0.0590 (8)	
O3	0.7449 (2)	0.69059 (9)	0.1815 (2)	0.0618 (8)	
H3A	0.6784	0.6982	0.2059	0.093*	
O4	0.7610 (2)	0.49241 (8)	0.0835 (2)	0.0627 (8)	
H4A	0.7009	0.4782	0.1067	0.094*	
O5	0.3253 (2)	0.65203 (10)	0.5037 (2)	0.0559 (7)	
O6	0.3497 (3)	0.57527 (10)	0.5661 (2)	0.0674 (8)	
O7	0.4132 (2)	0.55776 (9)	0.7805 (2)	0.0625 (8)	
H7A	0.3931	0.5512	0.7119	0.094*	
O8	0.3815 (3)	0.75593 (9)	0.8687 (3)	0.0688 (8)	
H8A	0.3502	0.7709	0.8122	0.103*	
O1W	0.5601 (4)	0.53969 (17)	0.4499 (5)	0.164 (2)	
H1A	0.5263	0.5510	0.3770	0.246*	
H1B	0.5107	0.5518	0.5061	0.246*	
O2W	0.4794 (19)	0.7748 (7)	0.1426 (18)	0.175 (8)	0.25
H2A	0.4951	0.7462	0.1748	0.262*	0.25
H2B	0.4471	0.7692	0.0728	0.262*	0.25
C1	0.5335 (3)	0.63324 (14)	0.2289 (3)	0.0428 (9)	
C2	0.6459 (3)	0.61142 (12)	0.1815 (3)	0.0374 (8)	
C3	0.7463 (3)	0.64152 (13)	0.1592 (3)	0.0438 (9)	
C4	0.8490 (3)	0.62084 (14)	0.1130 (3)	0.0527 (10)	
H4	0.9160	0.6405	0.0985	0.063*	
C5	0.8524 (3)	0.57144 (14)	0.0885 (3)	0.0532 (11)	
H5	0.9217	0.5581	0.0573	0.064*	
C6	0.7541 (3)	0.54164 (13)	0.1099 (3)	0.0451 (9)	
C7	0.6515 (3)	0.56179 (12)	0.1553 (3)	0.0410 (9)	
H7	0.5848	0.5418	0.1687	0.049*	
C11	0.3464 (3)	0.62091 (14)	0.5830 (4)	0.0452 (10)	
C12	0.3709 (3)	0.63929 (12)	0.7055 (3)	0.0379 (9)	
C13	0.4065 (3)	0.60753 (13)	0.7972 (4)	0.0440 (9)	
C14	0.4356 (3)	0.62616 (15)	0.9088 (4)	0.0543 (10)	
H14	0.4607	0.6050	0.9695	0.065*	
C15	0.4274 (3)	0.67550 (15)	0.9302 (4)	0.0535 (10)	
H15	0.4482	0.6876	1.0049	0.064*	
C16	0.3888 (3)	0.70707 (13)	0.8417 (4)	0.0441 (9)	
C17	0.3611 (3)	0.68917 (12)	0.7302 (3)	0.0417 (9)	
H17	0.3354	0.7107	0.6705	0.050*	
C21	0.0050 (3)	0.64614 (15)	0.4309 (4)	0.0552 (11)	
H21	0.0377	0.6773	0.4431	0.066*	
C22	−0.1138 (4)	0.63710 (16)	0.4643 (4)	0.0647 (12)	
H22	−0.1592	0.6619	0.4968	0.078*	
C23	−0.1624 (4)	0.59145 (16)	0.4486 (4)	0.0614 (12)	
H23	−0.2415	0.5848	0.4702	0.074*	
C24	−0.0925 (3)	0.55470 (14)	0.3999 (3)	0.0490 (10)	
C25	−0.1337 (4)	0.50485 (16)	0.3857 (4)	0.0598 (12)	
H25	−0.2114	0.4964	0.4080	0.072*	
C26	−0.0631 (4)	0.47059 (16)	0.3413 (4)	0.0586 (11)	
H26	−0.0931	0.4388	0.3329	0.070*	
C27	0.0583 (4)	0.48147 (14)	0.3058 (3)	0.0491 (10)	
C28	0.1355 (4)	0.44647 (14)	0.2590 (4)	0.0626 (12)	
H28	0.1086	0.4144	0.2484	0.075*	
C29	0.2502 (4)	0.45961 (14)	0.2292 (4)	0.0617 (12)	
H29	0.3022	0.4368	0.1978	0.074*	
C30	0.2876 (4)	0.50804 (13)	0.2466 (4)	0.0552 (11)	
H30	0.3660	0.5167	0.2266	0.066*	
C31	0.1027 (3)	0.52963 (13)	0.3183 (3)	0.0421 (9)	
C32	0.0266 (3)	0.56682 (13)	0.3680 (3)	0.0417 (9)	
C33	0.2558 (3)	0.74832 (14)	0.3709 (4)	0.0526 (10)	
H33	0.2890	0.7391	0.4446	0.063*	
C34	0.2328 (4)	0.79784 (15)	0.3495 (5)	0.0650 (13)	
H34	0.2506	0.8211	0.4076	0.078*	
C35	0.1838 (4)	0.81149 (14)	0.2419 (5)	0.0616 (12)	
H35	0.1677	0.8444	0.2261	0.074*	
C36	0.1574 (3)	0.77631 (13)	0.1546 (4)	0.0465 (10)	
C37	0.1035 (4)	0.78818 (15)	0.0403 (4)	0.0587 (12)	
H37	0.0841	0.8206	0.0220	0.070*	
C38	0.0809 (3)	0.75324 (16)	−0.0406 (4)	0.0614 (12)	
H38	0.0451	0.7617	−0.1139	0.074*	
C39	0.1107 (3)	0.70333 (14)	−0.0161 (4)	0.0455 (10)	
C40	0.0917 (3)	0.66543 (17)	−0.0998 (4)	0.0591 (11)	
H40	0.0586	0.6725	−0.1750	0.071*	
C41	0.1219 (3)	0.61860 (15)	−0.0698 (4)	0.0549 (11)	
H41	0.1090	0.5933	−0.1236	0.066*	
C42	0.1721 (3)	0.60929 (15)	0.0422 (4)	0.0496 (10)	
H42	0.1942	0.5772	0.0608	0.060*	
C43	0.1612 (3)	0.69012 (13)	0.0952 (3)	0.0386 (9)	
C44	0.1847 (3)	0.72736 (13)	0.1834 (3)	0.0386 (9)	

### Atomic displacement parameters (Å^2^)

**Table:**

	*U*^11^	*U*^22^	*U*^33^	*U*^12^	*U*^13^	*U*^23^
Cd	0.04016 (15)	0.03730 (16)	0.03675 (18)	−0.00085 (12)	0.00215 (11)	−0.00014 (13)
N1	0.0454 (17)	0.0433 (18)	0.043 (2)	−0.0004 (14)	0.0040 (15)	0.0050 (15)
N2	0.0491 (18)	0.0400 (17)	0.038 (2)	−0.0047 (14)	0.0013 (15)	0.0008 (15)
N3	0.0441 (17)	0.0397 (18)	0.039 (2)	−0.0014 (13)	0.0033 (15)	−0.0020 (16)
N4	0.0418 (16)	0.0407 (17)	0.0295 (18)	0.0013 (13)	0.0030 (14)	−0.0008 (15)
O1	0.0436 (15)	0.0530 (16)	0.071 (2)	0.0009 (12)	0.0142 (14)	0.0005 (14)
O2	0.0641 (17)	0.0426 (16)	0.071 (2)	0.0051 (13)	0.0099 (15)	−0.0185 (14)
O3	0.0644 (18)	0.0385 (15)	0.083 (2)	−0.0101 (12)	0.0099 (16)	−0.0104 (14)
O4	0.0762 (19)	0.0411 (16)	0.073 (2)	0.0051 (13)	0.0205 (16)	−0.0073 (14)
O5	0.0759 (19)	0.0534 (17)	0.0372 (17)	−0.0074 (14)	−0.0060 (14)	0.0017 (14)
O6	0.101 (2)	0.0471 (17)	0.055 (2)	−0.0132 (15)	0.0091 (17)	−0.0127 (15)
O7	0.083 (2)	0.0428 (16)	0.062 (2)	0.0002 (14)	0.0042 (16)	0.0066 (14)
O8	0.092 (2)	0.0461 (17)	0.067 (2)	−0.0010 (15)	−0.0033 (17)	−0.0163 (15)
O1W	0.106 (3)	0.200 (5)	0.191 (5)	0.021 (3)	0.043 (3)	0.052 (4)
O2W	0.22 (2)	0.158 (17)	0.144 (19)	0.026 (15)	−0.026 (16)	0.012 (14)
C1	0.042 (2)	0.049 (2)	0.037 (2)	0.0022 (18)	−0.0025 (17)	−0.0046 (19)
C2	0.0400 (19)	0.037 (2)	0.035 (2)	0.0011 (15)	0.0003 (17)	0.0003 (17)
C3	0.050 (2)	0.041 (2)	0.040 (3)	−0.0044 (16)	0.0013 (19)	0.0014 (17)
C4	0.047 (2)	0.054 (3)	0.058 (3)	−0.0085 (18)	0.012 (2)	0.009 (2)
C5	0.049 (2)	0.060 (3)	0.052 (3)	0.0049 (19)	0.014 (2)	0.001 (2)
C6	0.058 (2)	0.040 (2)	0.037 (2)	0.0063 (18)	0.0031 (19)	−0.0011 (18)
C7	0.042 (2)	0.039 (2)	0.042 (2)	−0.0003 (16)	0.0018 (18)	0.0032 (17)
C11	0.043 (2)	0.049 (3)	0.044 (3)	−0.0119 (17)	0.0054 (18)	−0.008 (2)
C12	0.0353 (19)	0.043 (2)	0.036 (2)	−0.0043 (15)	0.0035 (16)	0.0005 (17)
C13	0.048 (2)	0.039 (2)	0.046 (3)	−0.0022 (17)	0.0075 (19)	0.005 (2)
C14	0.057 (2)	0.060 (3)	0.046 (3)	−0.005 (2)	−0.003 (2)	0.014 (2)
C15	0.057 (2)	0.067 (3)	0.036 (3)	−0.009 (2)	0.002 (2)	−0.005 (2)
C16	0.048 (2)	0.045 (2)	0.039 (3)	−0.0036 (17)	0.0025 (19)	−0.005 (2)
C17	0.044 (2)	0.037 (2)	0.043 (3)	0.0012 (16)	0.0008 (18)	0.0035 (18)
C21	0.053 (2)	0.052 (2)	0.061 (3)	0.0064 (19)	0.010 (2)	0.006 (2)
C22	0.058 (3)	0.065 (3)	0.073 (3)	0.017 (2)	0.017 (2)	0.014 (2)
C23	0.045 (2)	0.083 (3)	0.057 (3)	0.010 (2)	0.012 (2)	0.024 (3)
C24	0.043 (2)	0.066 (3)	0.038 (2)	−0.0071 (19)	−0.0060 (19)	0.017 (2)
C25	0.049 (2)	0.080 (3)	0.049 (3)	−0.023 (2)	−0.007 (2)	0.020 (2)
C26	0.066 (3)	0.059 (3)	0.050 (3)	−0.027 (2)	−0.008 (2)	0.010 (2)
C27	0.063 (3)	0.046 (2)	0.036 (2)	−0.0118 (19)	−0.008 (2)	0.0047 (19)
C28	0.087 (3)	0.043 (2)	0.057 (3)	−0.012 (2)	−0.006 (3)	−0.001 (2)
C29	0.073 (3)	0.042 (2)	0.070 (3)	0.001 (2)	0.006 (2)	−0.007 (2)
C30	0.058 (2)	0.046 (2)	0.062 (3)	−0.0027 (19)	0.010 (2)	0.000 (2)
C31	0.049 (2)	0.044 (2)	0.032 (2)	−0.0084 (17)	−0.0033 (18)	0.0103 (18)
C32	0.042 (2)	0.054 (2)	0.029 (2)	−0.0024 (17)	−0.0011 (17)	0.0100 (18)
C33	0.055 (2)	0.048 (2)	0.055 (3)	−0.0009 (19)	0.001 (2)	−0.010 (2)
C34	0.069 (3)	0.045 (3)	0.081 (4)	0.000 (2)	0.005 (3)	−0.019 (3)
C35	0.054 (3)	0.036 (2)	0.095 (4)	0.0041 (18)	0.009 (3)	0.001 (3)
C36	0.036 (2)	0.043 (2)	0.061 (3)	0.0022 (17)	0.011 (2)	0.004 (2)
C37	0.050 (2)	0.049 (3)	0.078 (4)	0.0118 (19)	0.011 (2)	0.021 (3)
C38	0.051 (2)	0.076 (3)	0.058 (3)	0.010 (2)	0.006 (2)	0.030 (3)
C39	0.038 (2)	0.058 (3)	0.041 (3)	0.0046 (17)	0.0042 (18)	0.012 (2)
C40	0.051 (2)	0.088 (3)	0.039 (3)	−0.001 (2)	0.000 (2)	0.002 (3)
C41	0.054 (2)	0.070 (3)	0.041 (3)	−0.002 (2)	0.003 (2)	−0.010 (2)
C42	0.047 (2)	0.059 (3)	0.044 (3)	0.0043 (19)	0.006 (2)	−0.002 (2)
C43	0.0302 (18)	0.045 (2)	0.041 (2)	0.0007 (15)	0.0055 (17)	0.0082 (19)
C44	0.0310 (18)	0.043 (2)	0.043 (3)	0.0016 (15)	0.0098 (17)	0.0058 (19)

### Geometric parameters (Å, °)

**Table:**

Cd—O1	2.225 (2)	C14—H14	0.9300
Cd—O5	2.237 (3)	C15—C16	1.374 (5)
Cd—N1	2.360 (3)	C15—H15	0.9300
Cd—N4	2.389 (3)	C16—C17	1.379 (5)
Cd—N3	2.412 (3)	C17—H17	0.9300
Cd—N2	2.422 (3)	C21—C22	1.396 (5)
N1—C21	1.322 (4)	C21—H21	0.9300
N1—C32	1.360 (4)	C22—C23	1.361 (5)
N2—C30	1.327 (4)	C22—H22	0.9300
N2—C31	1.358 (4)	C23—C24	1.397 (5)
N3—C33	1.325 (4)	C23—H23	0.9300
N3—C44	1.353 (4)	C24—C32	1.410 (5)
N4—C42	1.319 (4)	C24—C25	1.439 (5)
N4—C43	1.366 (4)	C25—C26	1.332 (5)
O1—C1	1.270 (4)	C25—H25	0.9300
O2—C1	1.245 (4)	C26—C27	1.440 (5)
O3—C3	1.364 (4)	C26—H26	0.9300
O3—H3A	0.8200	C27—C28	1.402 (5)
O4—C6	1.380 (4)	C27—C31	1.405 (5)
O4—H4A	0.8200	C28—C29	1.364 (5)
O5—C11	1.252 (4)	C28—H28	0.9300
O6—C11	1.262 (4)	C29—C30	1.394 (5)
O7—C13	1.375 (4)	C29—H29	0.9300
O7—H7A	0.8200	C30—H30	0.9300
O8—C16	1.373 (4)	C31—C32	1.450 (5)
O8—H8A	0.8200	C33—C34	1.394 (5)
O1W—H1A	0.9410	C33—H33	0.9300
O1W—H1B	0.9244	C34—C35	1.360 (6)
O2W—H2A	0.8772	C34—H34	0.9300
O2W—H2B	0.8639	C35—C36	1.400 (5)
C1—C2	1.495 (5)	C35—H35	0.9300
C2—C7	1.390 (4)	C36—C44	1.404 (5)
C2—C3	1.405 (5)	C36—C37	1.434 (6)
C3—C4	1.387 (5)	C37—C38	1.339 (6)
C4—C5	1.378 (5)	C37—H37	0.9300
C4—H4	0.9300	C38—C39	1.424 (5)
C5—C6	1.380 (5)	C38—H38	0.9300
C5—H5	0.9300	C39—C43	1.399 (5)
C6—C7	1.377 (5)	C39—C40	1.414 (5)
C7—H7	0.9300	C40—C41	1.358 (5)
C11—C12	1.494 (5)	C40—H40	0.9300
C12—C17	1.396 (4)	C41—C42	1.383 (5)
C12—C13	1.395 (5)	C41—H41	0.9300
C13—C14	1.390 (5)	C42—H42	0.9300
C14—C15	1.373 (5)	C43—C44	1.443 (5)
			
O1—Cd—O5	101.93 (10)	C16—C17—H17	119.4
O1—Cd—N1	152.57 (10)	C12—C17—H17	119.4
O5—Cd—N1	87.41 (10)	N1—C21—C22	123.5 (4)
O1—Cd—N4	92.14 (10)	N1—C21—H21	118.3
O5—Cd—N4	151.96 (10)	C22—C21—H21	118.3
N1—Cd—N4	91.11 (10)	C23—C22—C21	119.0 (4)
O1—Cd—N3	113.72 (9)	C23—C22—H22	120.5
O5—Cd—N3	82.76 (10)	C21—C22—H22	120.5
N1—Cd—N3	92.88 (9)	C22—C23—C24	119.5 (4)
N4—Cd—N3	69.34 (10)	C22—C23—H23	120.2
O1—Cd—N2	83.18 (9)	C24—C23—H23	120.2
O5—Cd—N2	117.68 (10)	C23—C24—C32	118.0 (4)
N1—Cd—N2	69.74 (10)	C23—C24—C25	123.4 (4)
N4—Cd—N2	87.76 (10)	C32—C24—C25	118.6 (4)
N3—Cd—N2	151.29 (10)	C26—C25—C24	121.6 (4)
C21—N1—C32	118.0 (3)	C26—C25—H25	119.2
C21—N1—Cd	124.1 (3)	C24—C25—H25	119.2
C32—N1—Cd	117.9 (2)	C25—C26—C27	121.7 (4)
C30—N2—C31	117.9 (3)	C25—C26—H26	119.1
C30—N2—Cd	126.2 (2)	C27—C26—H26	119.1
C31—N2—Cd	115.9 (2)	C28—C27—C31	117.7 (4)
C33—N3—C44	118.5 (3)	C28—C27—C26	123.5 (4)
C33—N3—Cd	124.9 (3)	C31—C27—C26	118.8 (4)
C44—N3—Cd	116.6 (2)	C29—C28—C27	119.9 (4)
C42—N4—C43	117.3 (3)	C29—C28—H28	120.1
C42—N4—Cd	125.3 (3)	C27—C28—H28	120.1
C43—N4—Cd	117.5 (2)	C28—C29—C30	118.5 (4)
C1—O1—Cd	122.5 (2)	C28—C29—H29	120.7
C3—O3—H3A	109.5	C30—C29—H29	120.7
C6—O4—H4A	109.5	N2—C30—C29	123.7 (4)
C11—O5—Cd	120.0 (2)	N2—C30—H30	118.2
C13—O7—H7A	109.5	C29—C30—H30	118.2
C16—O8—H8A	109.5	N2—C31—C27	122.3 (3)
H1A—O1W—H1B	106.5	N2—C31—C32	118.1 (3)
H2A—O2W—H2B	106.5	C27—C31—C32	119.5 (3)
O2—C1—O1	124.3 (3)	N1—C32—C24	122.0 (3)
O2—C1—C2	119.3 (3)	N1—C32—C31	118.3 (3)
O1—C1—C2	116.3 (3)	C24—C32—C31	119.8 (3)
C7—C2—C3	119.0 (3)	N3—C33—C34	122.9 (4)
C7—C2—C1	121.0 (3)	N3—C33—H33	118.5
C3—C2—C1	119.9 (3)	C34—C33—H33	118.5
O3—C3—C4	119.3 (3)	C35—C34—C33	118.7 (4)
O3—C3—C2	121.5 (3)	C35—C34—H34	120.7
C4—C3—C2	119.2 (3)	C33—C34—H34	120.7
C5—C4—C3	120.6 (3)	C34—C35—C36	120.4 (4)
C5—C4—H4	119.7	C34—C35—H35	119.8
C3—C4—H4	119.7	C36—C35—H35	119.8
C4—C5—C6	120.6 (3)	C35—C36—C44	117.1 (4)
C4—C5—H5	119.7	C35—C36—C37	123.1 (4)
C6—C5—H5	119.7	C44—C36—C37	119.8 (4)
C7—C6—C5	119.3 (3)	C38—C37—C36	120.9 (4)
C7—C6—O4	121.9 (3)	C38—C37—H37	119.6
C5—C6—O4	118.8 (3)	C36—C37—H37	119.6
C6—C7—C2	121.2 (3)	C37—C38—C39	121.1 (4)
C6—C7—H7	119.4	C37—C38—H38	119.4
C2—C7—H7	119.4	C39—C38—H38	119.4
O5—C11—O6	124.4 (4)	C43—C39—C40	117.2 (4)
O5—C11—C12	117.6 (3)	C43—C39—C38	119.7 (4)
O6—C11—C12	118.0 (4)	C40—C39—C38	123.1 (4)
C17—C12—C13	118.4 (3)	C41—C40—C39	119.7 (4)
C17—C12—C11	120.4 (3)	C41—C40—H40	120.2
C13—C12—C11	121.2 (3)	C39—C40—H40	120.2
O7—C13—C14	118.5 (4)	C40—C41—C42	118.8 (4)
O7—C13—C12	121.7 (4)	C40—C41—H41	120.6
C14—C13—C12	119.8 (3)	C42—C41—H41	120.6
C15—C14—C13	120.6 (4)	N4—C42—C41	124.4 (4)
C15—C14—H14	119.7	N4—C42—H42	117.8
C13—C14—H14	119.7	C41—C42—H42	117.8
C14—C15—C16	120.3 (4)	N4—C43—C39	122.6 (3)
C14—C15—H15	119.8	N4—C43—C44	117.7 (3)
C16—C15—H15	119.8	C39—C43—C44	119.7 (3)
C15—C16—O8	117.6 (4)	N3—C44—C36	122.4 (3)
C15—C16—C17	119.7 (4)	N3—C44—C43	118.8 (3)
O8—C16—C17	122.7 (4)	C36—C44—C43	118.8 (4)
C16—C17—C12	121.2 (3)		

### Hydrogen-bond geometry (Å, °)

**Table:**

*D*—H···*A*	*D*—H	H···*A*	*D*···*A*	*D*—H···*A*
O1W—H1A···O1	0.94	2.09	2.974 (6)	155
O1W—H1B···O6	0.92	2.03	2.892 (6)	155
O2W—H2A···O2	0.88	1.99	2.869 (18)	175
O2W—H2B···O8^i^	0.86	2.42	3.28 (2)	173
O3—H3A···O2	0.82	1.81	2.540 (3)	147
O4—H4A···O7^ii^	0.82	2.09	2.877 (3)	160
O7—H7A···O6	0.82	1.82	2.546 (3)	147
O8—H8A···O3^iii^	0.82	2.10	2.917 (4)	171
C23—H23···O1W^iv^	0.93	2.49	3.339 (6)	153
C25—H25···O6^v^	0.93	2.50	3.285 (5)	143
C30—H30···O1	0.93	2.56	3.155 (5)	122
C33—H33···O5	0.93	2.50	3.105 (5)	123
C38—H38···O2^vi^	0.93	2.36	3.182 (5)	147
C42—H42···O4^vii^	0.93	2.58	3.231 (5)	127

Symmetry codes: (i) *x*, *y*, *z*−1; (ii) −*x*+1, −*y*+1, −*z*+1; (iii) *x*−1/2, −*y*+3/2, *z*+1/2; (iv) *x*−1, *y*, *z*; (v) −*x*, −*y*+1, −*z*+1; (vi) *x*−1/2, −*y*+3/2, *z*−1/2; (vii) −*x*+1, −*y*+1, −*z*.
